# Exploring Structure–Property Relationships in Aromatic Polybenzoxazines Through Molecular Simulation

**DOI:** 10.3390/polym10111250

**Published:** 2018-11-12

**Authors:** Scott Thompson, Corinne A. Stone, Brendan J. Howlin, Ian Hamerton

**Affiliations:** 1 Department of Chemistry, Faculty of Engineering and Physical Sciences, University of Surrey, Guildford, Surrey GU2 7XH, UK; scott.thompson@hexcel.com (S.T.); b.howlin@surrey.ac.uk (B.J.H.); 2 Dstl, Porton Down, Salisbury SP4 0JQ, UK; cstone@mail.dstl.gov; 3 Bristol Composites Institute (ACCIS), School of Civil, Aerospace, and Mechanical Engineering, University of Bristol, Queen’s Building, University Walk, Bristol BS8 1TR, UK

**Keywords:** polybenzoxazines, polymerization kinetics, thermal stability, molecular dynamics simulation

## Abstract

A series of commercial difunctional benzoxazine monomers are characterized using thermal and thermo-mechanical techniques before constructing representative polymer networks using molecular simulation techniques. Good agreement is obtained between replicate analyses and for the kinetic parameters obtained from differential scanning calorimetry data (and determined using the methods of Kissinger and Ozawa). Activation energies range from 85 to 108 kJ/mol (Kissinger) and 89 to 110 kJ/mol (Ozawa) for the uncatalyzed thermal polymerization reactions, which achieve conversions of between 85% and 97%. Glass transition temperatures determined from differential scanning calorimetry and dynamic mechanical thermal analysis are comparable, ranging from BA-a (151 °C, crosslink density 3.6 × 10^−3^ mol cm^−3^) containing the bisphenol A moiety to BP-a, based on a phenolphthalein bridge (239 to 256 °C, crosslink density 5.5 to 18.4 × 10^−3^ mol cm^−3^, depending on formulation). Molecular dynamics simulations of the polybenzoxazines generally agree well with empirical data, indicating that representative networks have been modelled.

## 1. Introduction

**Scheme 1:**
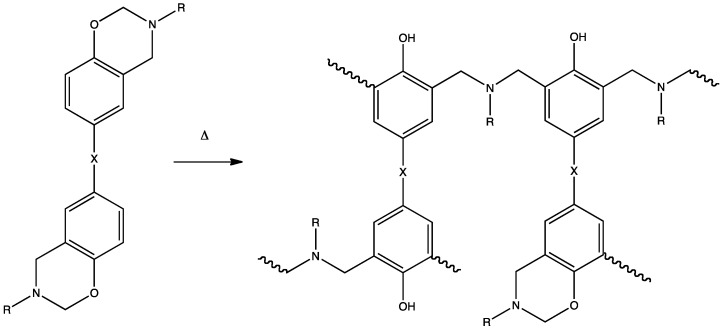
Bisbenzoxazines homopolymerization reaction showing ring opening and crosslinking.

Polybenzoxazines (PBZs) are modern thermosetting resins that are being explored [1] as possible replacements for common resins systems such as phenolic [2] and epoxy resins [3] in aerospace applications. When fully cured, PBZs offer a combination of favourable thermal and mechanical performance (e.g., dry *T*_g_ values of 246 °C, hot/wet *T*_g_ = 207 °C are possible [4], coupled with low moisture uptake), that gives an attractive property profile. Although requiring toughening for some engineering applications, PBZs can be combined with epoxy resins [5] or inherently engineering thermoplastics (e.g., oligomeric polyethersulphone, *K*_IC_ = 0.99 MPa m^1/2^) [6] to yield impressive enhancements. PBZs are formed through step-growth ring-opening polyaddition from *bis*-benzoxazine monomers (Scheme 1), which are in turn the products of the Mannich reaction between a bis-phenol, formaldehyde and a primary amine [7]. Unlike many other commercial thermosetting resins, which evolve condensation products such as water or ammonia, benzoxazine monomers react relatively cleanly to form a polymer with few reaction by-products [8], although the exact mechanism of the polymerization reaction to form a network has not been fully elucidated. Recent work in our group has examined the influence of additives on the nature of the polymerization mechanism, the formation of the polymer network structure and the resulting final properties [9].

The aim of the present paper is to use thermal, mechanical and computational techniques to explore structure-property relationships in a series of well-characterized commercial benzoxazine monomers and to use these data to inform the construction of representative networks using molecular simulation.

## 2. Experimental Section

### 2.1. Materials

**Figure 1:**
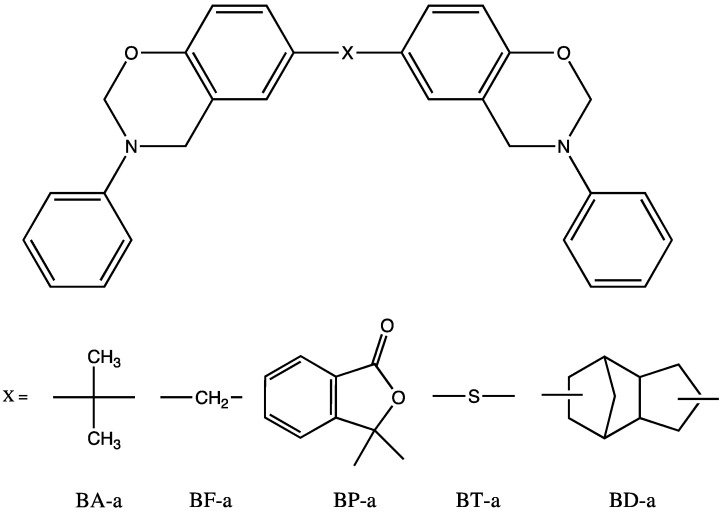
Structures of the monomers. *N.B.*, BF-a is a mixture of isomers: typically 4,4′-, 2,4′-, and 2,2′-BF-a in the approximate ratio 5:4:1 [10]). Monomer spectral data are deposited as supplementary data.

All monomers were supplied by Huntsman (Basel, Switzerland) and used as received. The relevant product names are Araldite MT35600 (based on the benzoxazine of bisphenol A and aniline, BA-a), MT35700 (based on the benzoxazine of a mixture of three isomers of bisphenol F and aniline, BF-a), MT35800 (based on the benzoxazine of phenolphthalein and aniline, BP-a, blended with BF-a), MT35900 (based on the benzoxazine of thiodiphenol and aniline, BT-a), LME10140 (based on the benzoxazine of tricyclo[5.2.1.^02,6^]decane-4,8-diol and aniline, BD-a), and LMB6490 (batch 2 representing BP-a, blended with butan-1-ol). All monomers (see Figure 1 for structures), except BP-a, were placed in a domestic baking silicone mould, degassed at approximately 90 °C for 1 hour using a vacuum oven to reduce void formation during the subsequent curing process. BP-a was found to be very difficult to control under degassing conditions (excessive void formation) and so was simply melted and held at 120 °C over the same time period that the other samples were degassed. Following degassing, all samples were placed in an air circulating oven at 120 °C and the following cure schedule was applied: heating 2 K min^−1^ to 180 °C (isothermal 2 hours) followed by heating at 2 K min^−1^ to 200 °C (isothermal 2 h).

### 2.2. Instrumentation

Differential scanning calorimetry (DSC) was performed on a TA Q1000 (TA Instruments, Crawley, West Sussex) with TA Q Series Advantage software on samples (4.0 ± 0.5 mg) in hermetically sealed aluminium pans. Samples were heated at 5, 8, 10, 12, and 15 K/min. from 25 to 300 °C (heat/cool/heat) in a nitrogen atmosphere (50 cm^3^/min.). Dynamic mechanical thermal analysis (DMTA) was performed on a TA Q800 in static air in single cantilever mode at a frequency of 1 Hz. Cured polymer samples (2 × 10 × 17 mm^3^) were scanned between −50 and 260 °C at 10 K/min.

### 2.3. Molecular Simulation

Molecular modelling was carried out using Materials Studio [11] (version 6.0, Accelrys, Cambridge, UK) on a PC. The potential energy for all models was calculated using the Condensed-phase Optimized Molecular Potential for Atomistic Simulation Studies (COMPASS) [12], a forcefield specifically designed for polymer calculations. Molecular models were all created using a combination of the Materials Studio modules and in-house programs to simulate a cure.

### 2.4. Construction of the Benzoxazines Using Molecular Mechanics

Initially, the structures of the benzoxazine monomers were simulated at zero Kelvin using molecular mechanics. Molecular mechanics (MM) [13] treats the molecule as an array of atoms governed by a set of classical-mechanical potential functions while the force field defines the mechanical model to represent the molecule. In order to optimize the structure of the molecule, the steepest-descents, conjugate gradients, and Newtonian [14] methods were performed to minimize the energy of the molecule. This yielded bond lengths that agreed well with published crystallographic data (Table 1) for similar monomers or compounds [15]. The Materials Studio Visualizer was used to create 3D models of the monomers, we then used 40 monomer models which were then packed into a periodic cell and repeated in a 3D space with voidless packing; the Ewald summation [16] was performed to neutralize the charges across the periodic boundaries.

### 2.5. Construction of the Cured Polybenzoxazines Using the Automated Cure Program

With the virtual space filled with the monomer mixture, control of the model was passed over to an in-house program to proceed with cure. A full discussion of the development of the automatic model building software falls beyond the scope of this paper, but it is outlined in more detail elsewhere [17]. The program selects and controls the reactions between benzoxazine groups to produce a realistic cured thermoset model. To model e.g., the polybenzoxazine based on BA-a the target density for the cell was set (within the automated program) at 1.15 g cm^−3^, consistent with the density found for the polymer (1.195 g cm^−3^) in the literature [18] and the monomers were packed to this value by the automated routine.

To form the cross-linked polymer network to different selected degrees of conversion, the automated curing program was employed with the polymer-consistent force field (PCFF) [19]. For the construction of the network, the automated program requires that a series of parameters be entered to determine the nature of the network formed. Thus, the cut-off distance (determining whether atoms were sufficiently close to undergo reaction) was set at between 5.5 and 7.5 Å depending on the experiment being formed; the dynamics duration was set at between 1000 and 10,000 fs and the simulated cure temperature was 453 K, reflecting the cure reaction.

### 2.6. Simulation of the Cured Polybenzoxazine Properties Using Molecular Dynamics

Temperature ramped MD simulations were carried out in the Temperature Cycle option in the Amorphous Cell Protocols. A constant-temperature, constant-pressure (NPT) ensemble (0.0001 GPa) was used at each experimental temperature, with a time step of 1 fs, employing the Anderson thermostat and Barendsen barostat [20]. COMPASS was used with the atomic van der Waals summation, a cut-off at 9.50 Å, a spline width of 1.00 Å and a buffer width of 0.50 Å. The Ewald summation [16] was performed to neutralize the charges across the periodic boundaries. MD simulations were carried out at different temperatures, with decrements of 10 K from the starting temperature (set at between 573 and 773 K), a total of 31 to 51 MD simulations were carried out, between 773 and 273 K. At each temperature stage the structure was minimized before the MD simulation to allow equilibration; the energies have practically reduced to a minimum after 25 ps and the simulation time was 200 ps.

## 3. Results and Discussion

### 3.1. Thermal Analysis of the Homopolymerization Reactions

**Figure 2:**
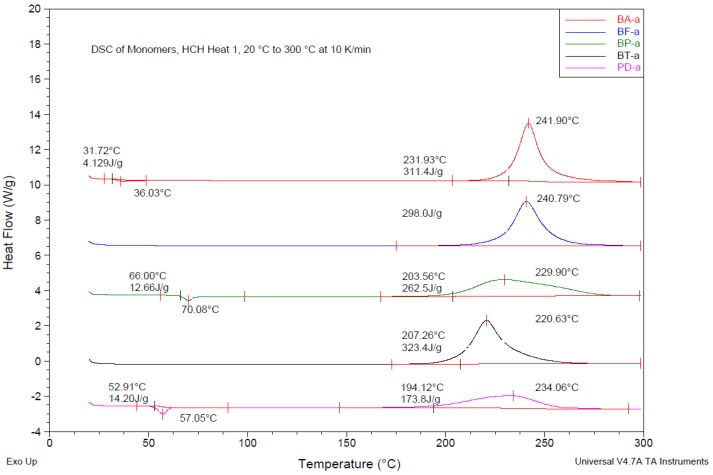
DSC of monomers, first heat of a heat cool heat cycle at 10 K/min.

The monomers were each analyzed using DSC to determine (a) the degree of cure and (b) the glass transition temperature (*T*_g_) for later use in the simulation study. In each case, the temperature program applied involved ‘heat-cool-heat’ with the sample being heated from room temperature to 300 °C at 10 K/min during each heat cycle. The DSC data produced from the first step of the heat, cool, heat cycle are presented in Table 1 (average of three measurements) with examples shown in Figure 2.

During the first heating cycle it was apparent that two of the five monomers (BP-a) and (BD-a) underwent very visible endothermic melting transitions. All of the monomers used in this work are solid at room temperature and one might expect to see these endotherms in all of the samples. What these data demonstrate is that it takes more energy for the BP-a and BD-a samples to melt, which may be due to a higher degree of crystallinity in these monomers. These benzoxazines, containing phenolphthalein and dicyclopentadiene moieties, respectively, have much bulkier bridging groups than those of the other three monomers, this may be the cause for this greater requirement of energy.

The exotherms, showing the polymerization reaction for all six monomers, display peak maxima within a narrow range of 218–242 °C. The monomers that display melting also show broad exotherms; the remaining monomers display much more pronounced peaks whilst curing. If a single exothermic peak is observed, then it is assumed that the curing results represent a single chemical process as a first approximation, although two or more simultaneous or very close chemical reactions cannot be ruled out [21] and previously we have used mathematical modelling to deconvolute the thermal data [9] to reveal contributions from several processes to the reaction exotherm. In addition, it has to be assumed that all of the heat generated is a result of the curing reaction, which is irreversible ring-opening and formation of the methylene bridge in the case of benzoxazines. The most symmetrical, Gaussian curve appears to be produced by the BF-a monomer with BA-a and BT-a appearing quite symmetrical, although there does appear to be slight trailing on the curve, perhaps suggesting that there is a small change in viscosity occurring in the system which has resulted in the reaction becoming more diffusion controlled. Both BP-a batches and BD-a form especially broad peaks representing a slower reaction and perhaps a combination of more than one reaction process occurring.

The lowest energy transition occurs for the BD-a sample at 183.5 J/g (50.9 kJ/mol Bz ring) and the highest, almost double, occurs at 326.0 J/g (73.8 kJ/mol Bz ring) for BT-a, based on the sulphur containing benzoxazine. The exotherm for BA-a (*T*_max_ ca. 240 °C and ∆*H*_p_ = 309.3 J/g) agrees reasonably closely with a literature value of *T*_max_ = 243 °C, although the enthalpy is significantly higher, ∆*H*_p_ = 240 J/g [22]. Pure benzoxazines typically show symmetrical exotherms between 200 and 250 °C, with ∆*H*_p_ = 150–600 J/g, although the exotherm may be skewed and reduced to lower temperatures in the presence of phenolic and amino impurities, arising from the synthesis of the monomer. In an extreme case, there will be multiple peaks, although none of the exotherms recorded here are truly multimodal.

### 3.2. Determination of Glass Transition Temperature Using DSC

**Figure 3:**
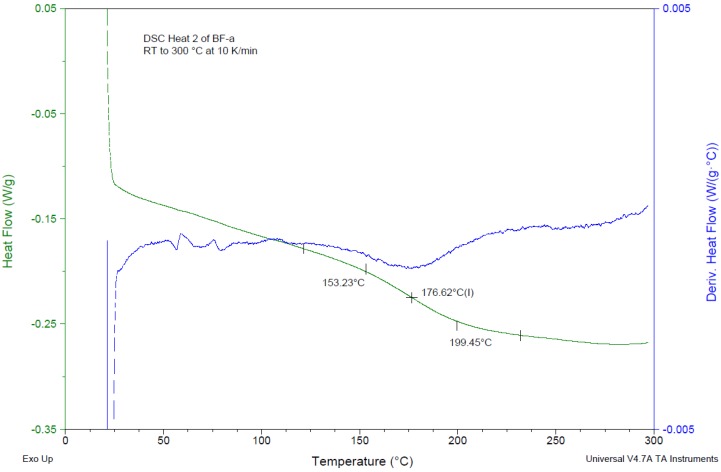
DSC, second heat of monomer BF-a demonstrating the *T*_g_ transition and analysis.

The second heating cycles of the DSC experiments were interpreted to yield the *T*_g_ of the monomers in their now cured resin form (Table 1). All the cured resins demonstrated a single *T*_g_, which strongly implies that the samples are homogeneous. The *T*_g_ values given here experimentally have been interpreted using the inflexion as exemplified by Figure 3.

The trend of the average glass transition temperature is: PBA-a < PBT-a < PBF-a < PBD-a < PBP-a, with PBP-a Batch 2 having the highest average *T*_g_ of all. This fits well with the knowledge that PBP-a Batch 2 and PBP-a have the same polybenzoxazine backbone. It is interesting to note that increasing the butanol content and decreasing the BF-a content of the BP-a formulation to create BP-a Batch 2 has resulted in a higher glass transition temperature by 17 K, presumably reflecting the increased reactivity (and higher crosslink density) of the system through a hydroxyl-initiated ring opening. The greatest *T*_g_ values found in this set of materials belong to the structures that contain the largest bisphenol linkages. The bulk of the dicyclopentadiene and phenolphthalein moieties no doubt restricts the molecular mobility of the chains with the ring structures limiting rotation. This will particularly be the case for the phenolphthalein group of PBP-a, which contains both a rigid aromatic ring and an oxygen atom, which may result in extra interactions with neighbouring chains (e.g., hydrogen bonding), which would also hinder chain motion and increase *T*_g_. When comparing the measured values to those available in the literature for the same monomer there is good agreement for PBA-a (151 °C measured, 150 °C literature [23]) and PBD-a (187 °C measured, 183 °C literature [24]).

### 3.3. Determination of Degree of Cure

DSC was also used to ascertain the degree of cure of the monomers by comparing the curing exotherm (first heat) with the corresponding polybenzoxazine sample that had been produced using a specific cure program. So, for instance: BA-a gave a cure exotherm of 309 J/g when cured to completion via DSC and was compared to the residual cure exotherm (31 J/g) of a sample cure in the laboratory using the cure program specified in the Experimental section, which was used throughout this work. Table 2 presents the resulting data, which demonstrate that in general the polybenzoxazines cure to a degree of approximately 90%.

### 3.4. Determination of Cure Kinetics Using DSC

**Figure 4:**
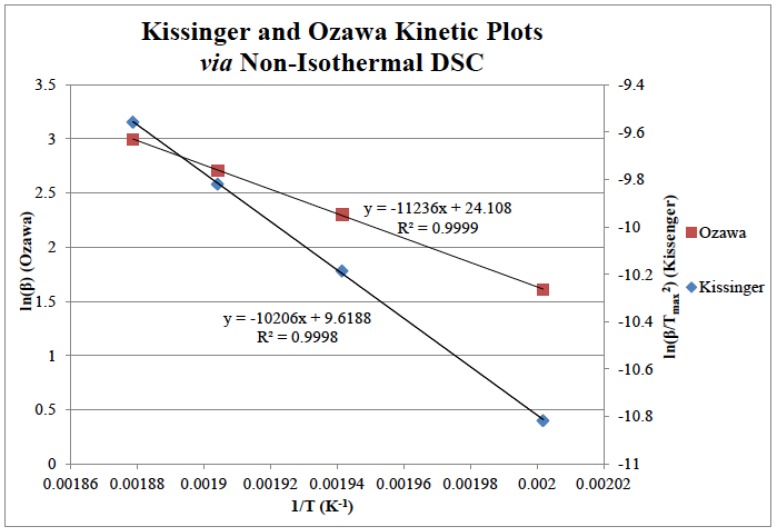
Kissinger and Ozawa plots for the polymerization of BA-a.

Two different methods of kinetic analysis, proposed independently by Kissinger and Ozawa, were used in this work, which use variations of the Arrhenius equation [25]. The Kissinger method is depicted in Equation (1):

(1) $$\;\ln\left(\frac{\beta }{T_{\max}^{2}}\right)=\ln\left(\frac{\mathrm{AR}}{E_{a}}\right)-\frac{E_{a}}{RT_{\max}^{2}}$$

where β = heating rate, *T*_max_ = temperature of DSC peak maxium, *E*_a_ = activation energy, *A* = pre-exponential (collision) factor, *R* = gas constant. and employs thermal data determined using DSC. When lnβ is plotted against reciprocal temperature (Figure 4) the determination of activation energy is derived from the gradient and the pre-exponential factor from the intercept (Table 3). The Ozawa method is similar to the Kissinger method, where the inverse relationship between the logarithm of heating rate to exothermic peak temperature in Equation (2) (also Figure 4) allows graphical determination of activation from the slope of the plot of lnβ/*T*^2^_max_ versus reciprocal temperature. The results are also presented in Table 3.

(2) $$\;\ln\beta =\ln\left(\frac{AE_{a}}{R}\right)-\mathrm{lnF}\left(\alpha \right)-5.331-1.052\;\left(\frac{E_{a}}{RT\max}\right)\;$$

where β = heating rate, *T*_max_ = temperature of DSC peak maxium, *E*_a_ = activation energy, *A* = pre-exponential (collision) factor, R = gas constant, and F(α) is a constant function.

All analyses using both of the aforementioned methods showed a good linear relationship between heating rate and exothermic peak temperature with R^2^ values no lower than 0.99 throughout.

The activation energies calculated by the two methods are very similar for each sample giving a degree of confidence in the results and the results for BA-a also match very closely to literature values (81 and 85 kJ mol^−1^ by Kissinger and Ozawa respectively. The similarity in activation energy of BA-a, BF-a and BT-a where only 1 kJ mol^−1^ separates both Kissinger and Ozawa values suggests that very similar cure processes occur in these materials. The values for BD-a are much greater at ~110 kJ mol^−1^ and greater still for BP-a at ~135 kJ mol^−1^, such a jump in activation energy suggests a change in the manner of cure. An obvious pattern to note is that BA-a, BF-a and BT-a all have small bisphenol linkages and have low activation energies, whereas the monomers with larger bisphenol linkages have the greater activation energies with the bulkiest linkage of BP-a having the greatest energy barrier. It is therefore easy to assume that the size of the bisphenol linkage has a significant effect on polymerization with larger groups hindering cure. This seems counterintuitive when one attempts to relate the kinetic information to degree of cure where PBD-a gives by far the greatest conversion value. It has therefore been found that polymerization with a large activation energy does not necessarily lead to a lower degree of cure when cured under standard conditions.

### 3.5. Determination of the Glass Transition Temperature Using Dynamic Mechanical Thermal Analysis

**Figure 5:**
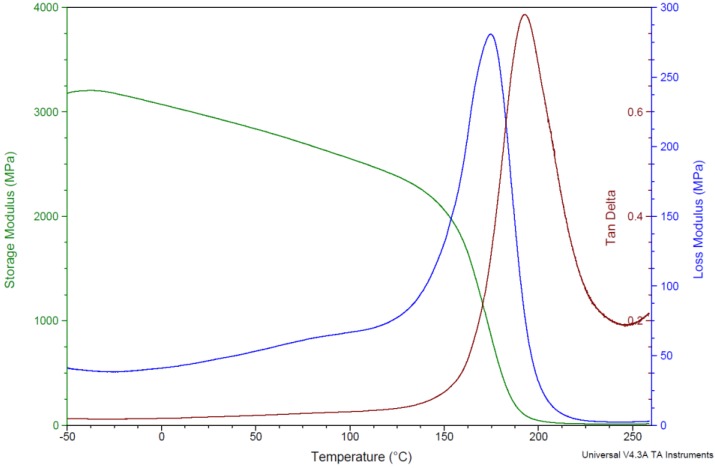
DMA plot of PBA-a (2 K/min from −50 °C to 200 °C at 0.1% strain, 1Hz FF).

DMTA is the primary method for determining the glass transition temperature (*T*_g_) of many polymers and has been identified to be several times more sensitive than DSC [26]. For this work a temperature range of −50 to 260 °C was used to allow identification of *T*_g_ and where possible β-transitions whilst remaining within the calibration range of the instrument. Figure 5 shows the DMTA plot produced for PBA-a from which a clear a clear *T*_g_ can be ascertained as the storage and loss moduli and tan δ change dramatically in the region of 125 to 240 °C as the polybenzoxazine loses its stiff, glassy nature, first becomes more plastic and ultimately more rubbery.

**Figure 6:**
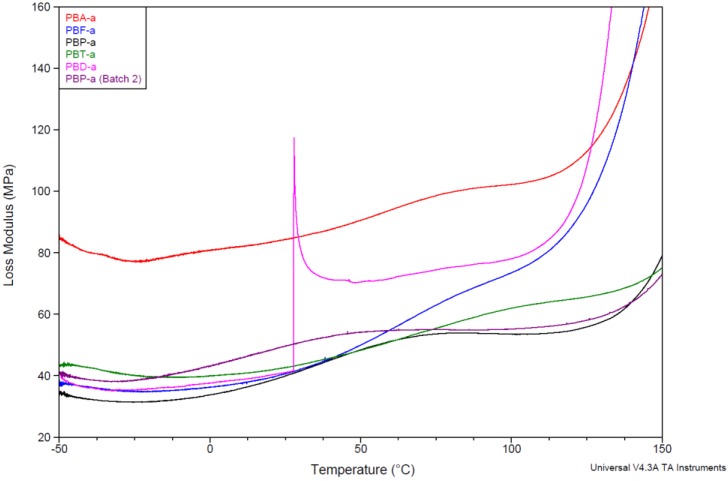
Loss Modulus of PBZs via DMA (2 K/min from −50 °C to 200 °C at 0.1% strain, 1Hz FF).

Figure 6 shows the portion of the DMTA plots, which reveal the β-transitions, which occurs in the range −25 to 120 °C with the PBT-a β-transition occurring at the highest temperature and PBP-a (Batch 2) occurring at the lowest. The β-transition allows a much more limited degree of movement and is usually localised to side-chains or branches from the main polymer backbone. In Figure 6 an even more restricted transition (γ-transition) can be seen at lower temperatures in some of the materials e.g., in PBA-a centered around 0 °C, with the other materials all showing what might be the end of this peak at −50 °C.

Table 4 presents the DMTA data for all of the polybenzoxazine materials used in this work, from which it is clear that the sulfur- and phenolphthalein-containing polybenzoxazine backbones give rise to greater values of *T*_g_ than their counterparts. The phenolphthalein backbone in both of its formulations gives the highest *T*_g_, with the ‘purified’ batch 2 version proving slightly superior (+3 K). This is to be expected as the Batch 1 sample contains a quantity of BF-a, which would depress *T*_g_. The order of *T*_g_ by DMTA then is: PBP-a > PBT-a > PBA-a > PBF-a > PBD-a with the PBD-a value being 20 K lower than the next lowest PBZ and PBP-a and PBT-a being 25 K higher than the next highest. It is difficult to postulate why this trend is apparent. PBA-a and PBF-a which have very similar backbones give close matching *T*_g_ values (within 7 K) of each other, whilst PBP-a and PBT-a match even more closely (within 2 K) whilst having markedly different bridging groups. When analyzed via differential scanning calorimetry (DSC) a similar trend in glass transition temperature was seen, however via DSC PBD-a gave the second highest *T*_g_ after PBP-a. It was suggested that this result showed a clear relationship between bisphenol linkage size and *T*_g_, where the large linkages would restrict molecular motion and increase *T*_g_. The change in PBD-a value when analyzed via DMA opposes this hypothesis as the materials with the two largest bisphenol linkages now give both the highest and lowest values for *T*_g_.

To gain a better understanding of these properties of the polybenzoxazines crosslink density has been calculated from the DMTA analyses using Equation (3). In theory, higher crosslink density of polymer networks can lead to increases in storage modulus and glass transition temperature (*T*_g_)^3^.

*G*_e_ = *ΦvRT*_e_ (3)

where *G*_e_ is the storage modulus at equilibrium, *Φ* is the front factor (unity for ideal rubbers), *R* is the gas constant, *v* is the crosslink density (number of moles of network chains per unit volume of cured polymer) and *T*_e_ is *T*_g_ + 50 °C [27].

The *T*_g_ data (Table 5) yield three groups of values: PBD-a by far the lowest, PBA-a and PBF-a intermediate and PBP-a and PBT-a the greatest, the same grouping can be seen in the crosslink density. PBD-a gives a crosslink density of 1.4 × 10^−3^ mol cm^−3^, less than half the value of PBA-a and PBF-a (3.6 and 3.8 × 10^−3^ mol cm^−3^ respectively) which are ca. 1.7 × 10^−3^ mol cm^−3^ below that of PBP-a and PBT-a. This is consistent with the accepted view that crosslink density has an influence on *T*_g_. As crosslink density increases chain movement/molecular mobility is further reduced resulting in more energy being required to overcome this obstacle. However, the results suggest that another factor may be at work as PBT-a has a greater crosslink density than PBP-a by 20% yet their *T*_g_ values only differed by 2 K. It has been suggested that free volume, hydrogen bonding, chain interaction and intermolecular packing can also influence these properties [28]. It is interesting to note how in the PBP-a, the crosslink density changes with removal of the BF-a (20%) in its formulation. The crosslink density of the PBP-a Batch 1 is 5.5 × 10^−3^ mol cm^−3^ compared with the 18.4 × 10^−3^ mol cm^−3^ of PBP-a Batch 2 has an increase of more than 300%. Particularly intriguing is that the large increase in crosslink density only results in a 3 K (1.5%) increase in *T*_g_, thus confirming that although crosslink density does influence *T*_g_ it is not the only influence.

### 3.6. Determination of the Glass Transition Temperature Using Molecular Dynamics Simulation

Molecular dynamics (MD) has been used to estimate *T*_g_ by simulating the location and velocity vector for each atom within a molecular model over time at specified conditions of temperature and pressure. The region representing the *T*_g_ is typically determined by performing simulation experiments at different temperatures and calculating the density of the model at each simulation temperature; *T*_g_ is estimated as the point of intersection between the thermal expansion gradients for higher and lower temperature data. In this work, the method reported by Hall et al. [17] was used to find the best point of gradient change (the ‘hinge point’) by finding when the fit quality of a line is at its maximum, based on finding the best fit for a gradient change as a function of temperature. An in-house program, written in Perl script, was used to analyze the raw data from the MD simulations, yielding a probability trace for *T*_g_, mapped against temperature. A peak position may represent the *T*_g_ and the breadth of the peak also indicates the overall quality of the simulation data. The ‘quality of fit’ is determined by centering an ellipse, of the same eccentricity as the standard deviation error bars and of sufficient radius to make a tangent with the best fit line.

**Figure 7:**
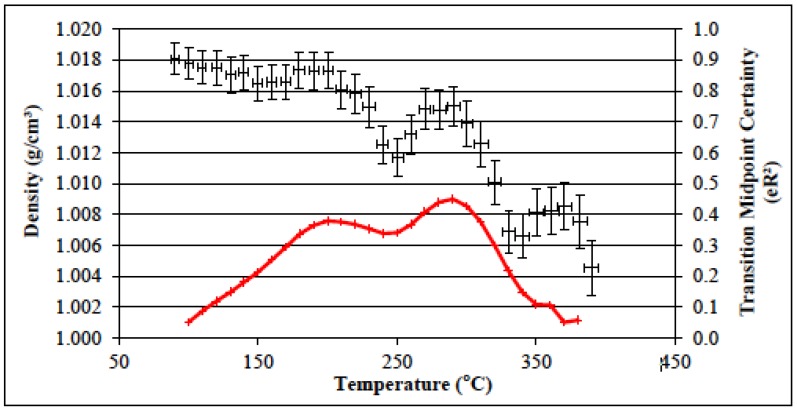
Plot of simulated density vs. temperature (black) for PBP-a with transition midpoint certainty aid (red).

A Beckerman box refinement method was employed to fit the line and minimize the total of the semi minor axis radii, which quantify the fit quality. Having calculated these parameters for a number of simulation temperatures, they were superimposed on the original density vs. temperature data. Examples of the calculations are shown on the simulated plots as the red line (the peak occurs where the gradient changes i.e., the transition midpoint certainty, TMC). Thus, the *T*_g_ is determined by the data rather than the experimentalist’s preconceived notion. A good example of this is shown for PBP-a (Figure 7) where two clear transitions are apparent, one over the range 190–240 °C and another above 290 °C. For comparison, the DMTA data for this material reveal an empirical *T*_g_ of 190–225 °C (Table 6). 

The second clear transition in the MD simulation for PBP-a is attributed to the onset of thermal degradation. Whilst a discussion of this phenomenon falls outside the scope of the present paper, this has been shown to correlate well with thermal stability data determined using thermogravimetric analysis [27].

**Figure 8:**
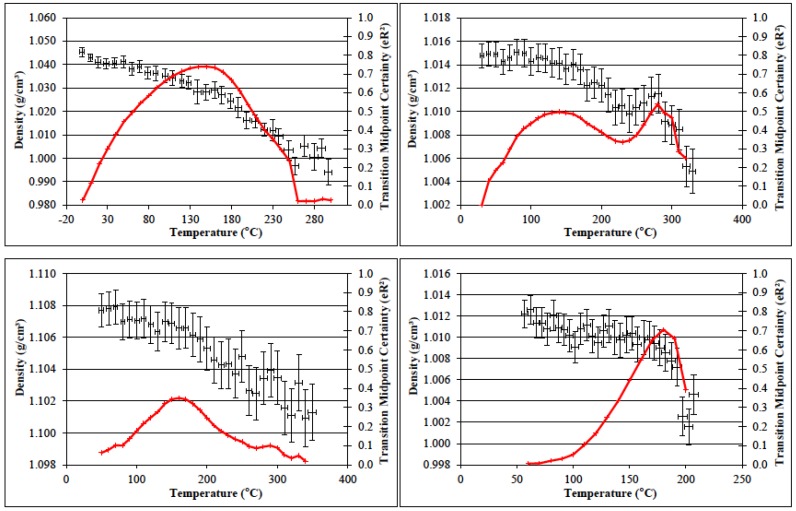
Plot of density vs. temperature of PBZs with TMC. PBA-a (**top left**), PBF-a (**top right**), PBT-a (**bottom left**), PBD-a (**bottom right**).

**Figure 9:**
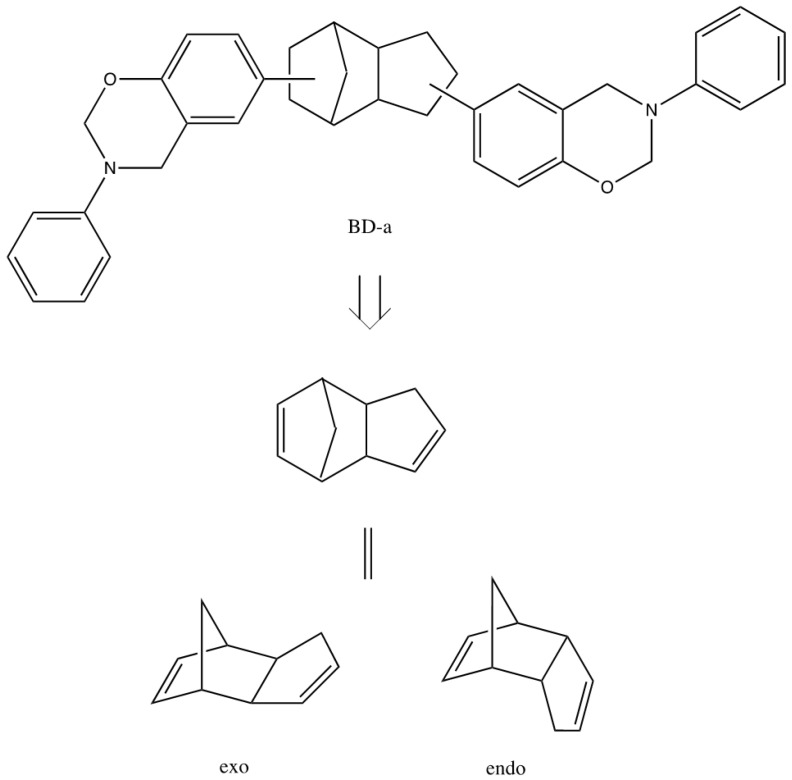
Structure of BD-a monomer, ultimately derived from dicyclopentadiene (showing *exo* and *endo* isomers).

Generally, the MD data show good agreement with the *T_g_* data produced using DMTA, but some simulated transitions are more easily discerned than others (Figure 8). For instance, in the case of PBA-a, the density starts to fall between 160 and 190 °C, which matches the empirical *T*_g_ for the same polymer measured by DMT; the TMC trace reveals a slightly lower value (150 °C). PBP-a shows a change in density between 190 and 220 °C (compared with a value of *T*_g_ of 200 °C determined using DMTA). The data for PBD-a are presented (Figure 8), but it is significantly harder to discern changes in the density plot, which is more uniform in the observed changes. A small change can be identified between 150 and 160 °C, but the TMC trace shows a maximum at 170 °C. This may suggest that the model for PBD-a is not a good representation (in terms of crosslink density and bulk density) of the authentic network. It may be that our simplified model does not capture the complexity of the isomeric mix. The parent benzoxazine would originally have been prepared commercially from dicyclopentadiene and a phenolic derivative via reaction at the C=C double bonds on each ring (the resulting bisphenyl molecule would have been subsequently reacted with aniline and formaldeyde (or paraformaldehyde) to yield the benzoxazine monomer [29]. Consequently, not only may the structural motif, which makes up the bridge of the monomer exists in both *exo* and *endo* forms [30] (Figure 9), but the initial reaction of dicyclopentadiene with the phenol derivative at either ends of the double bonds might have led to different isomers (or more likely different isomeric mixtures). These structural differences would all potentially lead to polymers for which the free volume and *T*_g_ values would vary, but further work is required to confirm this.

## 4. Conclusions

The DSC analyses presented here have allowed determination of a number of properties of the *bis*-benzoxazine monomers and their respective polybenzoxazines. The first heat of DSC analyses has demonstrated that both BP-a batches, BD-a and BA-a undergo a visible melting stage when heated suggesting that these materials are more crystalline than the other materials under study. The exotherms representative of ring-opening for all the monomers present peak maxima within a narrow range of 218–242 °C and that the polymerization enthalpy of the monomers is in the order BT-a > BA-a > BF-a > BP-a > BD-a. The degree of cure of the materials studied here is around 90% however PBD-a gives a value of 97% which is surprising given that BD-a contains a large bisphenol linking group which one might imagine would lower cure degree, which is seen for PBP-a. The second heat of the heat-cool-heat cycle has shown that the glass transition temperatures of the cured materials range from 150 to 256 °C in the order PBA-a < PBT-a < PBF-a < PBD-a < PBP-a. The nature of bisphenol linkage in the polybenzoxazine structure has a dramatic effect on the structural properties of the material with larger bisphenol linkages imparting greater *T*_g_s. Using the Kissinger and Ozawa methods activation energies of 80 kJ/mol^−1^ have been found for cure of BA-a, BF-a and BT-a with greater values of 110 and 135 kJ/mol^−1^ resulting for BD-a and BP-a respectively for the ring-opening polymerization process, with the bulkier bisphenol moieties yielding the greatest activation energies. Dynamic mechanical thermal analysis has revealed the glass transition temperature of the polybenzoxazines in this study to be in the order PBP-a > PBT-a > PBA-a > PBF-a > PBD-a in the range 147–204 °C. This suggests that the relationship between bisphenol linkage size and *T*_g_ is not as clear as had been seen via differential scanning calorimetry which gave rise to a much greater range in *T*_g_ between those of PBT-a and PBP-a. The crosslink density of the materials was also determined from DMTA data, with PBP-a Batch 2 demonstrating a crosslink density of 18.4 × 10^−3^ mol cm^−3^, almost triple the value of the next greatest polymer, PBT-a at 6.6 × 10^−3^ mol cm^−3^. The trend in crosslink density data in general matches well with the *T*_g_ data and molecular simulation has been used with some success to simulate these thermal events.

## Figures and Tables

**Table 1:** DSC First Heat Data of Monomers (20 to 300 °C at 10 K/min).

Monomer	*T*_m_ (°C)	∆*H*_m_ (J/g)	*T*_max_ (°C)	∆*H*_p_		*T*_g_ (°C)
				(J/g)	kJ/mol Bz	
BA-a	36	3	241	309	70	151
BF-a	-	-	240	298	65	174
BP-a	70	12	228	266	74	239
BP-a batch 2	80	7	234	239	66	256
BT-a	-	-	220	326	74	172
BD-a	57	14	234	184	51	187

Key: *T*_m_ = Peak Melt Temperature, ∆*H*_m_ = Enthalpy of Melt, *T*_max_ = Exothermic Peak Temperature, ∆*H*_p_ = Enthalpy of Polymerization.

**Table 2:** Degree of cure of polybenzoxazines.

PBZ	Cure exotherm (J/g)		Degree of Cure (%)
	Monomer	Cured Sample (rescan)	
PBA-a	309.30	31.11	89.9
PBF-a	298.30	23.24	92.2
PBP-a	265.37	24.08	90.9
PBP-a (Batch 2)	238.83	36.63	84.7
PBT-a	326.97	20.23	93.8
PBD-a	183.50	4.84	97.4

**Table 3:** Kinetic parameters for the polymerization of *bis*-benzoxazine monomers.

Monomer	Kissinger Method				Ozawa Method	
	*E*_a_ (kJ mol^−1^)	*k* × 10^−2^ (s^−1^)	*A* × 10^8^ (s^−1^)	R^2^ Value	*E*_a_ (kJ mol^−1^)	R^2^ Value
BA-a	85	21	1.53	0.9998	89	0.9999
BF-a	86	22	1.99	0.9995	90	0.9995
BP-a	135	50	559,425.15	0.9920	136	0.9929
BT-a	86	55	5.76	0.9998	90	0.9999
BD-a	108	34	602.97	0.9997	110	0.9997

Key: *k* = rate constant (s^−1^), *A* = pre-exponential factor (s^−1^).

**Table 4:** Glass transition temperature data produced via DMTA.

PBZ	Glass Transition Temperature (°C)		
	Storage Modulus	Loss Modulus	Tan δ
PBA-a	173	174	191
PBA-a (Batch 2)	175	176	195
PBF-a	166	167	186
PBF-a (Batch 2)	161	161	179
PBP-a	196	201	225
PBP-a (Batch 2)	202	204	230
PBT-a	197	199	213
PBD-a	146	147	164

**Table 5:** Crosslink density calculated from DMTA data.

Sample	*T*_e_ (K)	*G*_e_ (MPa)	*v* (×10^−3^ mol cm^−3^)
PBA-a	497	15.0	3.6
PBA-a (Batch 2)	498	15.2	3.7
PBF-a	490	15.4	3.8
PBF-a (Batch 2)	484	19.8	4.9
PBP-a	525	24.2	5.5
PBP-a (Batch 2)	527	80.8	18.4
PBT-a	522	28.7	6.6
PBD-a	470	5.4	1.4

Key: *T*_e_ is *T*_g_ + 50 °C, *G*_e_ is storage modulus at equilibrium, *v* is the crosslink density.

**Table 6:** Comparison of empirical *T*_g_ values with molecular dynamics simulations.

Sample	Glass transition temperature (°C)			
	Storage Modulus	Loss modulus	Tan δ	Simulation
PBA-a	173	174	191	160–190
PBF-a	166	167	186	170–190
PBP-a	196	201	225	190–220
PBT-a	197	199	213	180–220
PBD-a	146	147	164	150–160
